# Serial Changes of Quadriceps and Hamstring Muscle Strength Following Total Knee Arthroplasty: A Meta-Analysis

**DOI:** 10.1371/journal.pone.0148193

**Published:** 2016-02-05

**Authors:** Young-Wan Moon, Hyun-Jung Kim, Hyeong-Sik Ahn, Dae-Hee Lee

**Affiliations:** 1 Department of Orthopaedic Surgery, Samsung Medical Center, Sungkyunkwan University School of Medicine, Seoul, Korea; 2 Department of Preventive medicine, Korea University College of Medicine, Seoul, Korea; Georgia Regents University, UNITED STATES

## Abstract

This meta-analysis was performed to analyze serial changes in thigh muscles, including quadriceps and hamstring muscles, from before to one year after total knee arthroplasty (TKA). All studies sequentially comparing isokinetic quadriceps and hamstring muscle strengths between the TKA side and the contralateral uninjured limb were included in this meta-analysis. Five studies with 7 cohorts were included in this meta-analysis. The mean differences in the strengths of quadriceps and hamstring muscles between the TKA and uninjured sides were greatest three months after surgery (26.8 N∙m, 12.8 N∙m, P<0.001), but were similar to preoperative level at six months (18.4 N∙m, 7.4 N∙m P<0.001) and were maintained for up to one year (15.9 N∙m, 4.1 N∙m P<0.001). The pooled mean differences in changes in quadriceps and hamstring strengths relative to preoperative levels were 9.2 N∙m and 4.9 N∙m, respectively, three months postoperatively (P = 0.041), but were no longer significant after six months and one year. During the year after TKA, quadriceps and hamstring muscle strengths were lowest after 3 months, recovering to preoperative level after six months, but not reaching the muscle strength on the contralateral side. Relative to preoperative levels, the difference in muscle strength between the TKA and contralateral knees was only significant at three months. Because decrease of strength of the quadriceps was significantly greater than decrease in hamstring muscle strength at postoperative three months, early rehabilitation after TKA should focus on recovery of quadriceps muscle strength.

## Introduction

Quadriceps strength is a major determinant of physical function in patients who have undergone total knee arthroplasty (TKA).[1–5] Quadriceps weakness after TKA persists over a substantial period of time, and may not recover to the level in the uninvolved or healthy limb even after several years.[6–8] Therefore, interlimb differences in quadriceps strength may persist for months and years after unilateral TKA.[9]

Characterization of serial changes in quadriceps recovery after TKA, including determination of the time of minimal quadriceps strength after TKA and its recovery to preoperative status is important, despite the inherent preoperative quadriceps weakness due to severe osteoarthritis in the knee awaiting TKA.[10] The time course for the decrease and recovery of quadriceps muscle strength from before to after surgery may help in planning rehabilitation schedules for patients who undergo TKA, [11] because rapid recovery to an adequate level of quadriceps strength is key to functional performance.[4,12] Most previous studies, however, have evaluated postoperative quadriceps strength on the TKA side relative to the uninvolved side at a single time point, such as 3, 6, or 12 months after surgery.[7,11,13] In addition, few studies have evaluated the time course of recovery of hamstring muscle strength.

This meta-analysis was therefore designed to analyze serial changes in the strength of thigh muscles, including the quadriceps and hamstring muscles, from before to one year after TKA. Not only absolute strength, but postoperative change relative to preoperative muscle strength, between the TKA and uninvolved sides was evaluated at each time point. We hypothesized that the strengths of the quadriceps and hamstring muscles could be restored postoperatively to their preoperative levels but would not attain muscle strength on the contralateral side during the first year after unilateral TKA, and that quadriceps strength would decrease more than hamstring strength after unilateral TKA.

## Materials and Methods

This meta-analysis was performed according to the guidelines of the preferred reporting items for systematic reviews and meta-analysis (PRISMA) statement (S1 PRISMA Checklist).

### Data & Literature Sources

The study design was based on Cochrane Review Methods. Multiple comprehensive databases, including MEDLINE (January 1, 1976 to January 31, 2015), EMBASE (January 1, 1985 to January 31, 2015), the Cochrane Library (January 1, 1987 to January 31, 2015) and KoreaMed (June 1, 1958 to January 31, 2015), were searched for studies that compared isokinetic strengths of the quadriceps and/or hamstring muscles between the limb that underwent TKA and the contralateral uninjured limb. There were no restrictions on language or year of publication. Search terms used in the title, abstract, MeSH and keywords fields included "Arthroplasty" [tiab] OR "Replacements" [tiab] OR "Prosthesis" [tiab] AND "Knee" [tiab], and "Muscle Strength" [MeSH] OR "Muscle Contraction" OR "Isometric Contraction" [MeSH] OR "Quadriceps" [tiab] OR "Muscle Contractions" [tiab] OR "Muscular Contraction" [tiab] OR "hamstring" [tiab] OR "hamstrings" [tiab]. After the initial electronic search, relevant articles and their bibliographies were searched manually. Articles identified were assessed individually for inclusion.

### Study Selection

Study inclusion was decided independently by two reviewers, based on the predefined selection criteria. Titles and abstracts were read; if suitability could not be determined, the full article was evaluated. Studies were included in the meta-analysis if (1) they included patients who underwent unilateral TKA and were evaluated before and after surgery at various time points; (2) they reported direct comparisons of isokinetic concentric thigh muscle strengths, including the quadriceps and hamstring muscles of the TKA and contralateral uninjured sides, (3) they measured isokinetic thigh muscle strength with a dynamometer as maximal peak torque, (4) they measured isokinetic thigh strength from before surgery to ≥6 months postoperatively, (5) they fully reported the sample numbers as well as means and standard deviations of the isokinetic strength of thigh muscles, and (6) they used adequate statistical methods to compare muscle strengths on the two sides.

### Data Extraction

Two reviewers independently recorded data from each study using a predefined data extraction form. Any disagreement unresolved by discussion was reviewed by a third author.

Variables recorded included: (1) means and standard deviations of isokinetic muscle strength (maximal peak torque) of the quadriceps and hamstrings of the knee that underwent TKA and the uninjured knee; (2) sample size; and (3) angular velocity, a measure of maximal peak torque. If these variables were not mentioned in the articles, we contacted the study authors by email to request these data.

### Assessment of Methodological Quality

Two reviewers independently assessed the methodological quality of each study using the Newcastle-Ottawa Scale, [14] as recommended by the Cochrane Non-Randomized Studies Methods Working Group. For our purposes, the Newcastle-Ottawa Scale’s star system, which awards stars depending on level of bias, was adjusted to a scale that included only low (one star), high, and unclear bias. Each study was judged on three criteria: the selection of the study groups, the comparability of the groups and the ascertainment of either the exposure or outcome of interest for case-control or cohort studies. Any unresolved disagreements between reviewers were resolved by consensus or by consultation with a third investigator.

### Statistical Analysis

The current meta-analysis had two main outcomes. The first was the mean difference in isokinetic concentric strength (maximal peak torque) of the quadriceps and hamstring muscles on the TKA and uninjured sides at each time point, from preoperatively to 3 and 6 months and 1 year after TKA. The other main outcome was the mean difference in relative muscle strength compared with preoperative level between the TKA and uninjured limbs. Random-effects meta-analyses were used to pool these outcomes across the included studies, estimating weighted mean differences in thigh muscle strength and their change based on preoperative status between the two limbs and their associated 95% confidence intervals (CIs). Heterogeneity was determined by estimating the proportion of between-study inconsistencies due to actual differences between studies, rather than differences due to random error or chance, using the I^2^ statistic, with values of 25%, 50%, and 75% considered low, moderate, and high, respectively. All statistical analyses were performed using RevMan version 5.2 and Stata/MP 13.0.

## Results

### Identification of Studies

**Fig 1:**
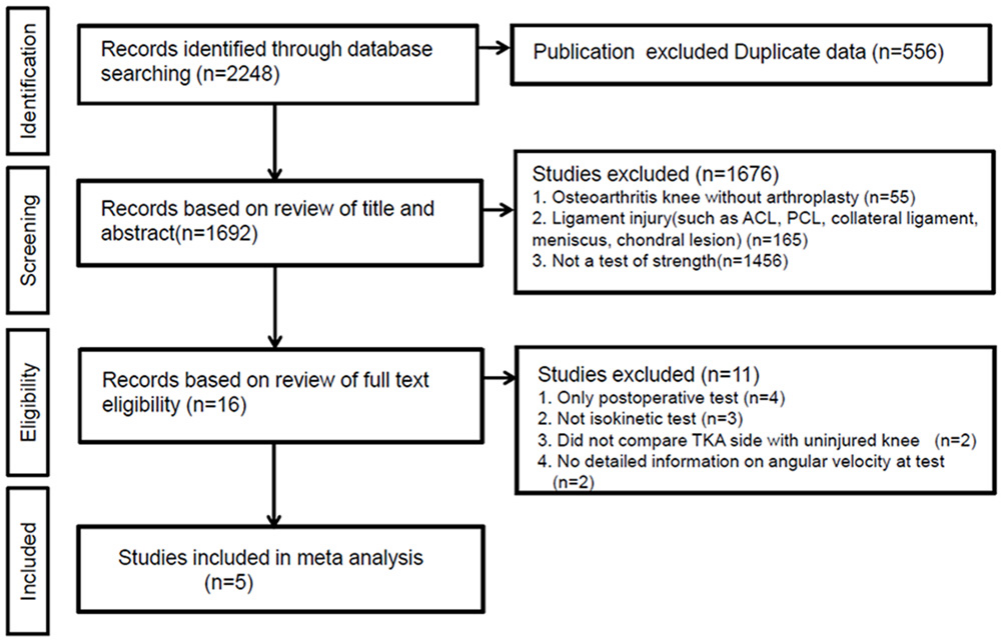
PRISMA (Preferred Reporting Items for Systematic reviews and Meta-analyses) flow diagram of the identification and selection of the studies included in this meta-analysis.

Fig 1 shows the details of study identification, inclusion, and exclusion. An electronic search yielded 912 studies in PubMed (MEDLINE), 1085 in EMBASE, 248 in the Cochrane Library and 27 in KoreaMed. Three additional publications were identified through manual searching. After removing 556 duplications, 1716 studies remained; of these, 1695 were excluded based on reading of the abstracts and full-text articles, and an additional 16 studies were excluded since they did not measure serial changes in muscle strength and/or utilized inappropriate measurement modalities such as isometric tests. After applying these criteria, 5 studies were finally included in this meta-analysis.

### Study Characteristics and Patient Populations

The 5 included studies included 7 patient cohorts and 274 knees of 274 patients who underwent unilateral TKA. Serial thigh muscle strengths, including those of the quadriceps and hamstring muscles, were measured in both legs from before to 6 months or 1 year after surgery. All five studies prospectively compared results on the TKA and uninjured sides. Three studies measured thigh muscle strength at 60°/sec of angular velocity; the fourth measured angular velocity at 90°/sec and 180°/sec; and the fifth measured angular velocity at 30°/sec and 120°/sec. All studies measured thigh muscle strengths preoperatively. Three studies measured strengths at 3 and 6 months and 1 year postoperatively; the fourth measured strengths at 6 months and 1 year, and the sixth measured strengths at 3 and 6 months (Table 1).

**Table 1:** Study characteristics.

Author	Year	Study type	Sample size	Measured Parameters (angular velocity)	Measured time points
Anchuela et al.[21]	2001	PCS	28	Q(60°/sec), H(60°/sec)	Preop, 6 Mo, 1 yr
Berman et al.[22]	1991	PCS	68	Q(60°/sec), H(60°/sec)	Preop, 3 Mo, 6 Mo, 1 yr
Lee et al.[23]	1999	PCS	34	Q (90°/sec, 180°/sec), H(90°/sec, 180°/sec)	Preop, 3 Mo, 6 Mo, 1 yr
Lorentzen et al.[24]	1999	PCS	30	Q (30°/sec, 120°/sec), H(30°/sec, 120°/sec)	Preop, 3 Mo, 6 Mo
Schroer et al.[25]	2010	PCS	50	Q(60°/sec), H(60°/sec)	Preop, 3 Mo, 6 Mo, 1 yr

Abbreviations: PCS, prospective comparison study; Q, quadriceps; H, hamstring; Preop, preoperative level

### Assessment of Methodological Quality

All five studies included in this meta-analysis were at low risk of selection bias. All compared injured legs that underwent TKA with contralateral uninjured legs as controls and provided detailed demographic data. None assessed possible confounding factors. Follow up duration was defined as adequate if muscle strength was measured at 1 year postoperatively. None of the included studies mentioned the percentage of patients evaluated, relative to all patients who underwent TKA at that institution. All studies included in this meta-analysis were deemed as having a high risk of bias in terms of adequacy of follow-up (Table 2).

**Table 2:** Risk of bias summary: review authors’ judgments about the risk of each bias item for each study.

Author	Representativeness of the cases	Selection of control	Ascertainment of exposure	Outcome of interest not present at start of study	Comparabilityof cohorts	Control for any additional factor	Assessment of outcome	Sufficient follow- up	Adequacy of follow up
Anchuela et al.[21]	−	−	−	−	−	+	+	−	+
Berman et al.[22]	−	−	−	−	+	+	+	−	+
Lee et al.[23]	−	−	−	−	−	+	+	−	+
Lorentzen et al.[24]	−	−	−	−	−	+	+	+	+
Schroer et al.[25]	−	−	−	−	−	+	+	−	+

−, low risk of bias;

+, high risk of bias

### Isokinetic Quadriceps and Hamstring Strengths

**Fig 2:**
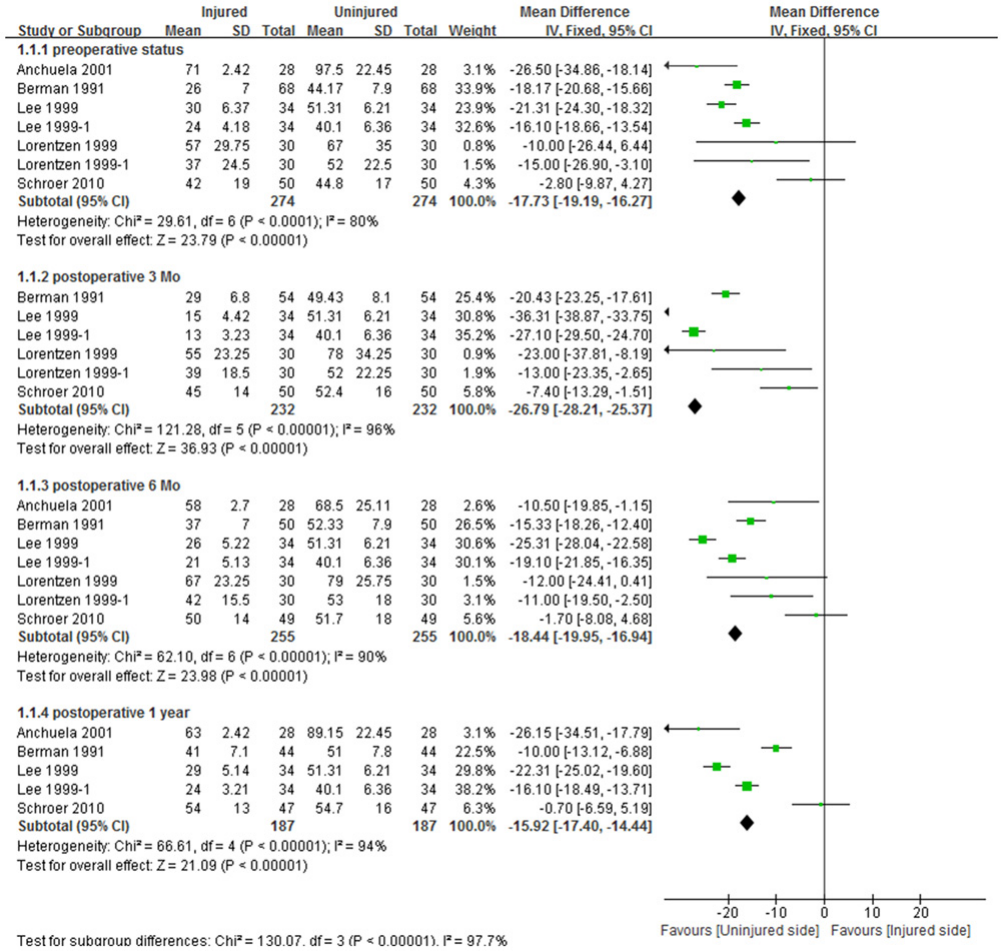
Forest plot demonstrating significant reductions in quadriceps strength on the TKA side relative to the uninjured limb from before to one year after surgery.

**Fig 3:**
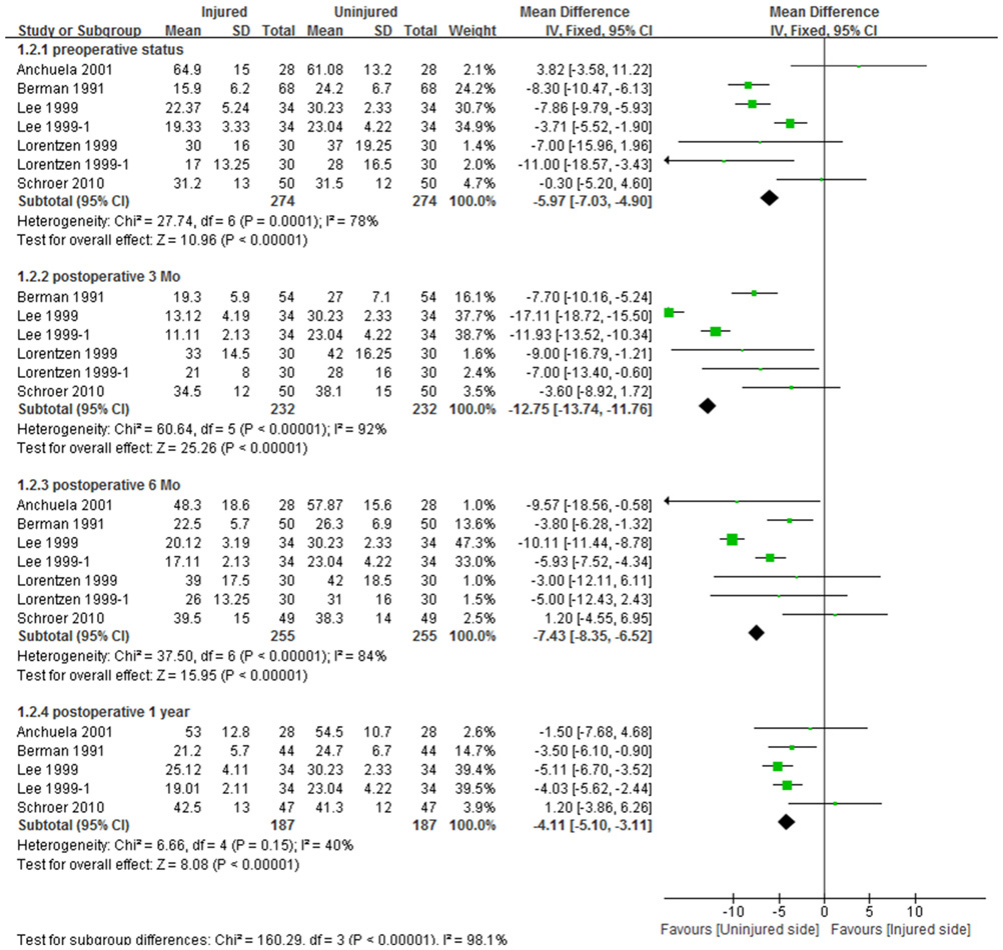
Forest plot showing significant reductions in hamstring strength on the TKA side relative to the uninjured limb from before to one year after surgery.

Five studies, including 7 comparison cohorts, compared the isokinetic strengths of quadriceps muscles of the TKA and uninjured legs. The pooled standard mean difference in quadriceps muscle strength on the two sides was 17.7 N∙m preoperatively (95% CI:16.3 to 19.2; P<0.001), indicating that isokinetic quadriceps strength was lower on the TKA than on the uninjured side. This mean difference between limbs was greatest 3 months after surgery (26.8 N∙m, 95% CI:25.4 to 28.2; P<0.001), but was similar to preoperative level at 6 months (18.4 N∙m, 95% CI:16.9 to 20.0; P<0.001) and was maintained for up to 1 year (15.9 N∙m, 95% CI:14.4 to 17.4; P<0.001, Fig 2). Similarly to the quadriceps, mean hamstring muscle strength was 6.0 N∙m lower (95% CI:4.9 to 7.0; P<0.001) on the TKA than on the uninjured side preoperatively. This mean difference between limbs was maximal 3 months after surgery (12.8 N∙m, 95% CI:11.8 to 13.7; P<0.001), recovering to preoperative levels after 6 months (7.4 N∙m, 95% CI:6.5 to 8.4; P<0.001) and maintained for up to 1 year (4.1 N∙m, 95% CI:3.1 to 5.1; P<0.001, Fig 3). The pooled mean difference in quadriceps strength between the two sides was approximately twice that of the mean difference in hamstring strength.

### Changes in muscle strength

**Fig 4:**
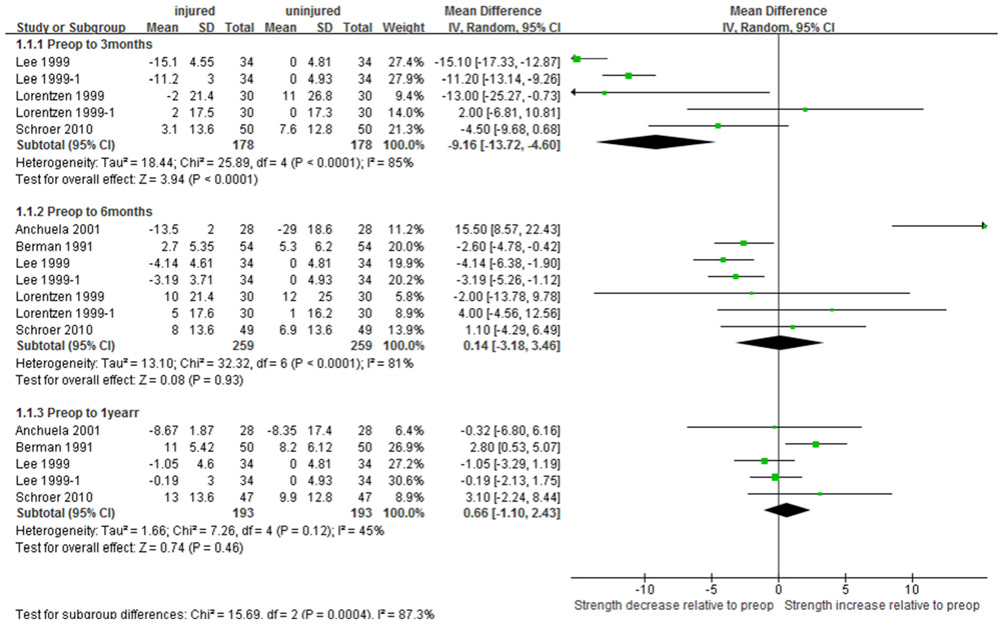
Forest plot demonstrating differences in quadriceps strength based on preoperative level between the TKA side and the uninjured limb.

**Fig 5:**
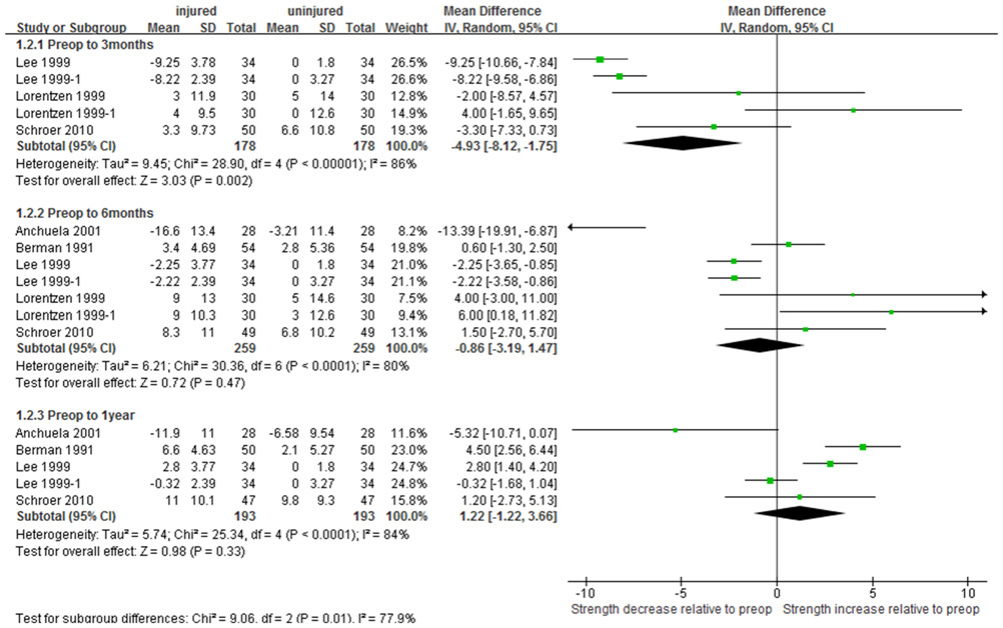
Forest plot demonstrating differences in hamstring strength based on preoperative level between the TKA side and the uninjured limb.

The pooled mean difference in quadriceps strength relative to preoperative level was -9.2 N∙m 3 months after TKA (95% CI: -13.7 to -4.6; P<0.001), but strengths relative to preoperative level were not significantly different at 6 months (0.14 N∙m, 95% CI: -3.2 to 3.5; P = 0.93) and 1 year (0.66 N∙m, 95% CI:-1.1 to 2.4; P = 0.46, Fig 4). Similarly, the pooled mean difference in hamstring strength relative to preoperative level was -4.9 N∙m 3 months after TKA (95% CI:-8.1 to -1.8; P<0.001), or about half of the change observed in the quadriceps muscle. Hamstring strengths relative to preoperative level were no longer significant at 6 months (-0.86, 95% CI:-3.2 to1.5; P = 0.47) and 1 year (1.22, 95% CI:-1.22 to3.7; P = 0.33) after TKA (Fig 5). The pooled mean decrease in muscle strength at postoperative 3 months relative to preoperative level was found to be 4.30 N∙m greater for quadriceps than hamstring muscle (P = 0.041). However, the pooled mean changes in muscle strength at postoperative 6 months and 1 year relative to preoperative level did not differ significantly between quadriceps and hamstring muscles (Table 3).

**Table 3:** Comparisons of the pooled mean changes in muscle strength between quadriceps and hamstring muscles at postoperative 3 and 6 months, and 1 year relative to preoperative levels.

	Quadriceps		Hamstring		
	Mean	SD	Mean	SD	P-value
Preop. to 3 months.	-9.2	21.97	-4.9	17.44	0.041
Preop. to 6 months	0.14	27.5	-0.86	19.29	0.632
Preop. to 1 year	0.66	12.4	1.22	3.7	0.548

## Discussion

The most important findings of this study were that quadriceps and hamstring muscle strengths showed a similar time course after TKA, with maximal decreases observed at 3 months, restoration to preoperative status at 6 months, and maintenance of preoperative level at 1 year. However, the decreases in quadriceps muscle strength were two- to three-fold greater than those of hamstring muscles.

Optimizing rehabilitation after TKA requires measurements of serial postoperative changes in quadriceps and hamstring strengths.[15,16] Previous studies evaluating sequential loss and recovery of quadriceps and hamstring strength have reported that the strength of both muscles decreased markedly during the early postoperative period and recovered with time, but did not reach the strengths of the contralateral uninvolved side even years after surgery [6,17]. Our findings were similar, suggesting that the strengths of both muscles decreased 3 months after TKA, recovered to their preoperative levels at 6 months, and were maintained for at least 1 year. However, the relative loss of strength in these muscles has varied across studies. For example, a recent evaluation of postoperative quadriceps and hamstrings muscle strength after TKA found no differences between these muscles in loss or recovery during the first 6 months after TKA [17]. In contrast, our current meta-analysis found that the decrease in muscle strength was approximately two- to three-fold greater in the quadriceps than in the hamstring muscle from before to 1 year after surgery. Specifically, the difference in strength 3 months after surgery relative to preoperative levels between the TKA and uninvolved sides was two-fold greater in quadriceps (8.9 N∙m) than in hamstring (4.7 N∙m) muscles. This discrepancy may have been due to differences in methods used to measure muscle strength. The former study [17] used isometric measurement tests, whereas our meta-analysis only included studies that used isokinetic tests. Although these two modalities are frequently used interchangeably to evaluate muscle strength in clinical settings, [18] they may estimate muscle weakness differently. Isometric tests may underestimate quadriceps weakness compared with isokinetic tests [18], which may eliminate any significant differences in loss of strength between the quadriceps and hamstrings muscles until 6 months after TKA.

This meta-analysis included only those studies that compared knees that underwent unilateral TKAs with contralateral knees (within subject comparisons).[18] Between-subject comparisons, in which subjects undergoing TKA are compared with healthy controls, require normative data, including sex, age, anthropometric characteristics and physical activity level, with the latter having a considerable impact on muscle strength.[18] Another confounding factor potentially affecting the validity of between-subject comparisons is pain and fear of pain, which are difficult to control.[19] Within-subject, side-to-side comparisons are therefore more practical for patients who undergo unilateral TKA, provided the uninvolved side is asymptomatic and not affected by severe OA.

This meta-analysis included evaluations of serial changes in recovery of muscle strength after TKA, not only by comparing absolute values at each time point but the differences based on preoperative muscle strengths on both side. Quadriceps and hamstring muscle strengths on both sides showed the greatest decrease 3 months after TKA, recovering later. The changes in muscle strength relative to preoperative level were significantly different between the TKA and uninvolved sides only at 3 months, although the absolute muscle strengths were lower on the TKA than on the contralateral side at all time points. The change in quadriceps strength from before to 3 months after TKA was about twice that of the hamstring during the same period. Although these findings suggest that quadriceps weakness may be greater than hamstring weakness at 3 months, these changes in muscle strength relative to preoperative status were similar on the two sides at 6 months and 1 year. Thigh muscle strengths after 6 months and 1 year were similar to preoperative levels, but were lower than in the contralateral uninvolved knee.

The results of this meta-analysis indicate that rehabilitation protocols should focus on quadriceps strengthening during the early postoperative period (3 months after TKA). Even after 3 months, thigh muscle strengthening exercises should be performed for recovery of muscle strength on the TKA side, thus minimizing interlimb differences in muscle strength.

This study had several limitations. First, this meta-analysis assumed that the uninvolved side is healthy. This may not be the case, at least for individuals who undergo TKA, many of whom have experienced some osteoarthritic changes on the contralateral side.[20] In addition, subjects involved in the studies included in this meta-analysis differed in type of surgery, use of prostheses, and rehabilitation protocols. This may have affected serial changes of muscle strength and increased the heterogeneity of included studies. However, heterogeneity was minimized by measuring muscle strengths using isokinetic tests and comparing muscle strengths using a within-subject approach.

## Conclusions

In conclusion, quadriceps and hamstring muscle strengths showed the greatest decreases 3 months after TKA, recovering to preoperative levels after 6 months, but not reaching the strength on the contralateral side. Decrease in quadriceps muscle strength was significantly greater than decrease in hamstring muscle strength at postoperative three months. Therefore, early rehabilitation after TKA should focus on recovery of quadriceps muscle strength.

## Supporting Information

S1 PRISMA Checklist PRISMA checklist. (PDF)
